# Evaluation of offspring sex ratio, sex hormones and antioxidant enzymes following exposure to methyl tertiary butyl ether in adult male Sprague-Dawley rats

**DOI:** 10.17179/excli2014-580

**Published:** 2015-01-13

**Authors:** Leila Khalili, Soghra Gholami, Maryam Ansari-Lari

**Affiliations:** 1 Department of Basic Sciences, School of Veterinary Medicine, Shiraz University, Shiraz, PO Box 1731, Postal code 71345, Iran; 2 Department of Food Hygiene and Public Health, School of Veterinary Medicine, Shiraz University, Shiraz, PO Box 1731, Postal code 71345, Iran

**Keywords:** Methyl tertiary butyl ether (MTBE), sex ratio, sex hormones, antioxidant enzymes

## Abstract

Methyl tertiary butyl ether (MTBE) is an oxygenated fuel additive which has been used widely in many parts of the world. This experiment was performed to determine the effect of MTBE on offspring sex ratio, sex hormones and antioxidant enzymes. A total of 20 adult Sprague-Dawley male rats were divided into four groups and received 0, 400, 800 and 1600 mg/kg/day MTBE by gavages for 30 consecutive days. At the end of the experiment, blood samples were taken for determination of sex hormones and antioxidant enzymes. Then, male rats were mated with healthy unexposed female rats and sex of offspring was determined after birth. Sex ratio was 0.48, 0.50, 0.43 and 0.50 in 0, 400, 800 and 1600 mg/kg/day MTBE groups, respectively (P = 0.91). There was significant decreasing trend for luteinizing hormone (LH) and testosterone in experimental groups (r_s_ = -0.50, P = 0.030 and r_s_ = -0.67, P = 0.002, respectively). No changes were observed for superoxide dismutase. However, decrease in glutathione peroxidase (GPX) was observed in all treatment groups compared with control which was significant in 400 mg/kg/day MTBE group (P = 0.016). The present study showed that paternal exposure to oral MTBE has no effect on offspring sex ratio; while, MTBE exposure could exert dose-dependent changes in serum testosterone and LH in treatment groups. The results of the present study, need to be clarified in the future studies.

## Introduction

Methyl tertiary butyl ether (MTBE) is an oxygenated fuel additive which has been used widely in many parts of the world. MTBE reduces air pollution by increasing the oxygen content of gasoline and decreasing carbon monoxide and other hydrocarbon emissions (Stern and Kneiss, 1997[20]). MTBE can migrate faster and farther in the ground than other gasoline components. This makes it more likely to contaminate public water systems and people could be exposed to MTBE by drinking, swimming, or showering in water contaminated with MTBE.

Based on several studies on carcinogenicity of MTBE in animals, it has been shown that chronic exposure to MTBE is associated with renal tubular cell tumors, hepatocellular adenomas and Leydig cell tumors of the testes in rodents (Bird et al., 1997[8]; Clegg et al., 1997[9]; Belpoggi et al., 1995[3]). Therefore, it has been listed as a potential human carcinogen (USEPA, 1998[21]). Based on the results of carcinogenicity studies in male and female rats, it has been suggested that MTBE might interfere with normal hormonal feedback mechanisms (Williams et al., 2000[22]).

In contrast to MTBE carcinogenicity, there are a few recently published studies of potential endocrine effects of MTBE, and associated male hormonal effects have not been addressed in detail. Furthermore, the studies on male reproductive hormones had inconsistent results. For example increase, decrease and no changes in testosterone levels have been reported in previous studies (Day et al., 1998[11]; Williams et al., 2000[22]; Billiti et al., 2005[7]; Li et al., 2008[16]; Bermudez et al., 2012[4]). On the other hand, one interesting aspect of reproduction is the sex ratio of offspring at birth. Considering the growing body of knowledge about the association of offspring sex ratio with many factors including environmental exposures (Rosenfeld and Roberts, 2004[18]; Mocarelli et al., 2000[17]; James, 1996[13]), there is no study concerning the effects of MTBE exposure in male on the offspring sex ratio at birth. It has been shown that paternal exposure to gasoline (containing MTBE) in filling station workers was associated with decrease in offspring sex ratio (Ansari-Lari et al., 2004[1]). Also, sex ratio was skewed to female births in Sprague-Dawley rats exposed to gasoline vapor for 6 hours for 30 consecutive days (Ansari-Lari and Tanideh, 2009[2]). However, to the best knowledge of the authors, studies which have addressed the association of MTBE exposure with offspring sex ratio were conducted either on female animals after conception and during gestation (Conaway et al., 1985[10]; Bevan et al., 1997[5]), or on both male and female animals prior to mating with continuous exposure of females during pregnancy (Biles et al., 1987[6]). 

Furthermore, there are very limited data about the role of oxidative stress in reproductive effect of MTBE (Li et al., 2007[15], 2008[16], 2009[14]). It has been shown that MTBE increased the production of reactive oxygen species (ROS) and enhanced lipid peroxidation in isolated rat spermatogenic cells (Li et al., 2007[15]), can exert reproductive system toxicity of male rats and disturb the secretions of sex hormones possibly due to induction of oxidative stress (Li et al., 2008[16]) and also has a direct toxic effect on cultured rat Sertoli cells (Li et al., 2009[14]). 

Taken together, the objective of the present study was to determine the effect of oral administration of MTBE on offspring sex ratio at birth, changes in sex hormones and two antioxidant enzymes, superoxide dismutase (SOD) and glutathione peroxidase in adult male Sprague-Dawley rats.

## Materials and Methods

A total of 20 adult Sprague-Dawley male rats weighing 223 ± 20 gr were purchased from animal house of “Sepid Exir Azma”, Shiraz, Iran. Animals were housed in stainless steel cages under standard animal house conditions with a 12 hr light/dark cycle and a temperature of 25 ± 2 °C, received standard pellet food, and tap water was available *ad libitum*. The experimental animals were randomly divided into four equal experimental groups after 10 days of acclimation period which received 0, 400, 800 and 1600 mg/kg/day MTBE in almond oil by gavages for 30 consecutive days. MTBE was prepared from Oil Refinery, Shiraz, Iran with 98.8 % purity. The study was approved by our institutional review board. Body weight and group food consumption were measured every week. At the end of the exposure period, male rats were anesthetized with ether and blood samples were individually obtained from heart. For determination of sex hormones, blood samples were collected in 2 ml micro-centrifuge tubes and serum was separated by centrifugation at 3000 rpm for 10 min and stored at -20 °C until use. For measurement of antioxidant enzymes activities, blood samples were collected in EDTA tubes. After blood sampling, animals in all experimental groups were mated with 44 healthy unexposed female rats. There were two females and one male rat in each cage for mating; one male in each group was mated with three female. In 800 mg/kg/day MTBE group, one animal was expired due to inappropriate gavages. Therefore, in this group three male were mated with three females each. Pregnant female rats were kept under standard housing conditions until the end of the pregnancy. Gender of offspring was determined after birth. 

### Serum sex hormones

Serum sex hormones including total testosterone (DRG ELISA kit, Germany), luteinizing hormone (Cusabio ELISA kit, PRC) and follicle-stimulating hormone (Cusabio ELISA kit, PRC) were determined using enzyme linked immunosorbent assay kits according to the manufacturer recommendations. 

### Antioxidant enzyme activities

Antioxidant enzyme including super oxide dismutase (SOD) and glutathione peroxidase (GPX) activities were determined by chemical methods using EDTA whole blood samples via appropriate kits (SOD and GPX kits, Biorexfars, Iran) as instructed by manufacturer.

### Statistical analysis

Data were presented as mean, median and standard deviation (SD). Sex hormones and antioxidant enzymes activities as well as litter size were compared between groups using nonparametric analysis of variance (Kruskal-Wallis tests) followed by Mann-Whitney U tests; Spearman’s rho correlation coefficient was used to determine their dose-response relationship. Offspring sex ratio at birth (expressed as male proportions) was determined for individual male rats in each group. After arc-sinus transformation, mean of group sex ratio was compared using one-way analysis of variance. Changes in body weight and food consumption during the study period were analyzed using repeated measurements analysis of variance. Fertility index was calculated in two ways: as number of fertile male to all males and also as number of pregnant females to all mated females in each group; statistical comparison between experimental groups was conducted using Chi-square for trend analysis. Data were analyzed using SPSS statistical software (Version 16.0; SPSS, Inc., Chicago, USA) and a P-value less than 0.05 was considered statistically significant in all analyses. 

## Results

Mean body weight was increased from 223 ± 20 gr at the start of the study to 281 ± 30 gr at the end of the experiment. There was significant increasing linear trends for body weight during the study period (P < 0.001) with no statistical difference between experimental groups (P = 0.40). Mean group food consumption was 169 ± 32 gr in the first week and was increased to 349 ± 28 in the last week of the study (P < 0.001). No adverse clinical observation was detected in experimental animals. 

Overall, 149 male and 155 female were produced by experimental groups. No stillbirth or gross anomaly was detected in experimental groups. Summary statistics for reproductive parameters are displayed in Table 1(Tab. 1). No significant difference between groups sex ratio (P = 0.91) or number of pups at birth (P = 0.11) was detected (Table 1(Tab. 1)). Fertility index based on pregnant females was equal to 81.8 % for the first three experimental groups and 54.5 % for 1600 mg/kg MTBE group (P = 0.22). When fertility index was calculated based on fertile males, it was 100 % for the first three groups and 60 % for 1600 mg/kg MTBE group (P = 0.058).

**Figure 1:**
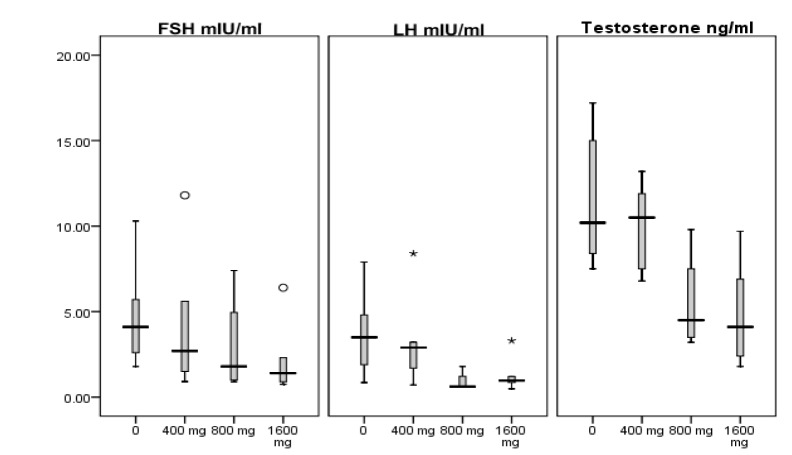
Box plots showing minimum, maximum and quartiles of serum luteinizing hormone (LH), follicle stimulating hormone (FSH) and testosterone levels, in MTBE-treated adult Sprague-Dawley male rats. Circles and asterisks show outliers and extreme values, respectively.

Comparisons of sex hormones between study groups are presented in Table 2(Tab. 2). There was no significant difference (P = 0.33) between study groups for level of follicle-stimulating hormone (FSH). Luteinizing hormone (LH) was significantly lower in 800 mg/kg/day (P = 0.032), and testosterone was lower in 1600 mg/kg/day MTBE group compared with control one (P = 0.032). Spearman’s rho correlation coefficient for levels of serum FSH, LH and testosterone with ordered dose groups were -0.43 (P = 0.064), -0.50 (P = 0.030) and -0.67 (P = 0.002), respectively. These decreasing trends with increasing dose (negative dose-response relationship) are clearly depicted in Figure 1(Fig. 1) which shows the quartiles of data in each experimental group.

Levels of antioxidant enzyme activities in different experimental groups are shown in Table 2(Tab. 2). No changes were observed for SOD in this study. However, decrease in glutathione peroxidase (GPX) was observed in all treatment groups compared with control one which was significant in 400 mg/kg/day MTBE group (P = 0.016). Furthermore, a tendency for significance (P = 0.09) was detected between 1600 mg/kg/day MTBE and control group (Table 2(Tab. 2)). No dose-response relationship was detected for antioxidant enzymes.

## Discussion

To the best knowledge of the authors, there is the first report about the effect of MTBE exposure of male rats on their offspring sex ratio. We found three studies which has addressed the association of MTBE exposure with offspring sex ratio. In the first study, no treatment related change was observed in fetal sex distribution when mated CD Sprague-Dawley rats and CD-1 mice were exposed during the period of organogenesis to target concentrations of 0, 250, 1000, and 2500 ppm MTBE (Conaway et al., 1985[10]). In the second study again with no effect on offspring sex ratio, both male and female rats were exposed by inhalation to 300, 1300 and 3400 ppm for 6 hours/day, 5 days/week before mating (12 weeks and 3 weeks, respectively) and then throughout mating and gestation (Biles et al., 1987[6]). The third study reported that following exposure of female pregnant mice in gestational day 6-15 to various doses of MTBE by inhalation, altered sex ratio as decreasing male fetuses was observed (Bevan et al., 1997[5]). Results of the present study showed that exposure of male rats to oral MTBE has no effect on their offspring sex ratio at birth (Table 1(Tab. 1)).

Previous studies have shown that paternal exposure to gasoline (containing MTBE) in filling station workers was associated with decrease in offspring sex ratio (Ansari-Lari et al., 2004[1]). Also, sex ratio was skewed to female births in offspring of male Sprague-Dawley rats exposed to gasoline vapor for 6 hours daily for 30 consecutive days (Ansari-Lari and Tanideh, 2009[2]). Based on the present results, it seems that changes in sex ratio may be attributed to some gasoline components other than MTBE. However, further studies are needed to clarify this finding.

Fertility was apparently lower in the 1600 mg/kg/day MTBE compared with other experimental groups, but did not show significant difference. It is suggested that high dose of MTBE has the potential to negatively affect the fertility in male Sprague-Dawley rats and it was not significant due to limited sample size. This finding need to be investigated in studies concerning changes in sperm quantity and quality in MTBE exposed male rats.

The number of pups at birth seems to be more related to female than male reproductive potential, to produce proper number of growing follicles in each cycle. Due to using healthy unexposed female rats in this study, the non-significant change in litter size is expectable. 

Studies on sex hormones including testosterone, LH and FSH in association with MTBE exposure in rats are limited and showed inconsistent results (Day et al., 1998[11]; Williams et al., 2000[22]; Billiti et al., 2005[7]; Li et al., 2008[16]; Bermudez et al., 2012[4]). In the present study, we did not observe significant changes in FSH, but a significant lower LH in 800 mg/kg/day MTBE compared with control one was observed. Also, a significant decrease in testosterone in 1600 mg/kg/day MTBE group compared with control group was detected which was in accordance with decrease in fertility in the same treatment group. In addition, we found significant dose-related decreases for LH and testosterone, and tendency to significance for FSH in the present study (Figure 1(Fig. 1)). Previous researchers observed a decrease in serum LH and testosterone at treatment level of 800 and 1500 mg oral MTBE/kg/day for 15 or 28 days (Day et al., 1998[11]; Williams et al., 2000[22]) as well as decreasing level of LH and FSH in rats exposed to gasoline inhalation for 30 days (Ansari-Lari and Tanideh, 2009[2]), which are consistent with our results. However, Li et al. (2008[16]) reported that serum levels of LH and FSH was increased significantly in male rats when exposed to 800 and 1600 mg/kg/day oral MTBE for 15 days and level of testosterone was significantly higher in a group which received 800 mg/kg MTBE by gavage for 28 when compared with the control group. 

Control of sex hormones has a complex mechanism. In theory, altered hepatic metabolism of testosterone (Williams et al., 2000[22]), direct effect of MTBE on hypothalamic-pituitary-gonadal axis (de Peyster et al., 2003[12]), or its direct effect on testosterone secreting cells in testis may be responsible for endocrine changes observed in MTBE exposed groups. But the other possible mechanism may be the role of genetic factors in the host response to toxic environmental exposures. Such a mechanism was suggested in a previous study who indicated that decrease in testosterone level in male gasoline filling station workers was most pronounced in the group with the glutathione S-transferase T1 (*GSTT1*) null genotype compared with the group possessing the *GSTT1* genotype (Saadat and Monzavi, 2008[19]). Therefore, the observed alterations in sex hormones following MTBE exposure merit further evaluation via investigating the genetic background of the host in future research.

In our present experiment, decrease in glutathione peroxidase (GPX) was observed in all treatment groups compared with control which was significant in 400 and near to significant in 1600 mg/kg/day MTBE groups (Table 2(Tab. 2)). No alteration in SOD and no dose-response relationship were detected in the present work. Increased maleic dialdehyde and total antioxidant ability as well as increased mRNA level of the extracellular form of SOD has been reported by Li et al. (2008[16]). They have suggested that high doses of MTBE (1600 mg/kg/day) could induce oxidative stress and may be responsible for reproductive toxicity and disturbance in hormonal secretions in male rats (Li et al., 2008[16]). No similar study for measuring blood SOD and GPX activities was found in the literature for comparison. However, decreasing GPX in MTBE treatment groups indicate the exposure of animal to oxidative stress, which may lead to oxidative damage in exposed groups. 

In conclusion, the present study showed that paternal exposure to various doses of oral MTBE for 30 days is not associated with offspring sex ratio at birth but has the potential to negatively affect the fertility in male Sprague-Dawley rats. MTBE exposure could exert negative effect on serum testosterone, LH and GPX activity in treatment groups. A dose-related decreasing pattern was observed for the effect of MTBE exposure on sex hormones, whereas the observed effect on GPX did not show dose-dependent trend. The results of the present study, need to be clarified in the future studies may be in conjunction with investigating the genetic background of the host. 

## Acknowledgement

This study was supported by Shiraz University.

## Conflict of interest

Authors declared that there is no conflict of interest.

## Figures and Tables

**Table 1:**
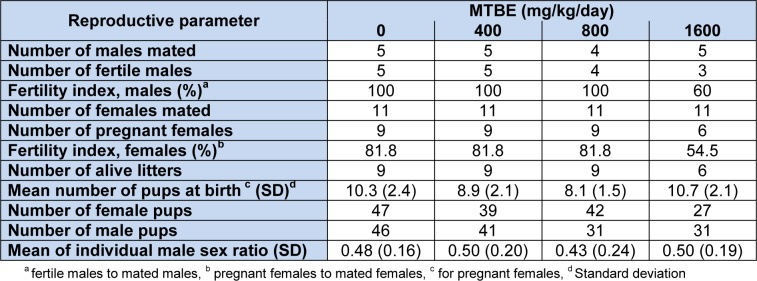
Summary statistics for reproductive outcomes in MTBE-treated adult Sprague-Dawley male rats

**Table 2:**
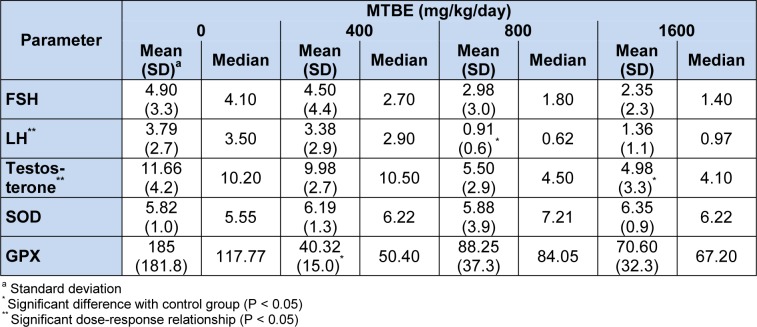
Comparison of serum luteinizing hormone (LH, mIU/ml), follicle stimulating hormone (FSH, mIU/ml), testosterone (ng/ml), superoxide dismutase (SOD, U/gHb) and glutathione peroxidase (GPX, U/gHb) in MTBE-treated (mg/kg/day) adult Sprague-Dawley male rats
